# Antibacterial secondary metabolites from an endophytic fungus, *Arthrinium* sp. MFLUCC16-1053 isolated from *Zingiber cassumunar*

**DOI:** 10.1080/21501203.2018.1481154

**Published:** 2018-05-31

**Authors:** Acharavadee Pansanit, Patcharee Pripdeevech

**Affiliations:** School of Science, Mae Fah Luang University, Chiang Rai, Thailand

**Keywords:** Antibacterial, antioxidant, *Arthrinium*, endophyte, GC-MS, *Zingiber cassumunar*

## Abstract

Forty-four endophytes were isolated from *Zingiber cassumunar* and identified morphologically. The ethyl acetate extracts of all endophytes were obtained. The ethyl acetate extracts were subjected to study antibacterial and antioxidant activities. The ethyl acetate extract of the *Arthrinium* sp. MFLUCC16-1053 showed activity against both gram-positive and gram-negative bacteria. Specifically, the minimum inhibition concentration against *Staphylococcus aureus* and *Escherichia coli* was 31.25 and 7.81 µg/mL, respectively. In addition, the crude extract showed highest antioxidant activity in 2,2-diphenyl-1-picrylhydrazyl scavenging at IC_50_ value of 28.47 µg/mL. Gas chromatography-mass spectrometry analysis revealed that the extract of the *Arthrinium* sp. MFLUCC16-1053 contains various antibacterial and antioxidant compounds which are β-cyclocitral, 3*E*-cembrene A, laurenan-2-one, sclareol, 2*Z*,6*E*-farnesol, cembrene, β-isocomene and γ-curcumene. The fungus *Arthrinium* sp. MFLUCC16-1053 was also identified by molecular and phylogenetic methods.

## Introduction

Endophytes or endophytic fungi are fungi isolated from plant tissues. They colonise inter- and intra-cellular areas of host plant tissues without causing disease. The relationship between host plant and endophytes is mutualistic benefiting both partners (Kogel et al. 2006). Endophytes may contribute to protecting their host plant from diseases by producing a diverse array of secondary metabolites such as terpenoids, palmarumycins, furandiones, phenols, dimeric anthrone and benzopyroanone (Schulz et al. 2002; Porras-Alfaro and Bayman 2011). Moreover, endophytes comprise a promising rich source of potential bioactive medicinal constituents such as podophyllotoxin (Canel et al. 2000), paclitaxel (Taxol) (Stierle et al. 1993), hypericin (Kusari et al. 2008) and camptothecin (Kusari et al. 2009). Generally, medicinal plants used in traditional medicine are selected for surveying bioactive endophytic fungi due to documented beneficial applications (Kaul et al. 2012; Kusari et al. 2013) and thus, high likelihood of producing pharmaceutically important secondary metabolites.

*Zingiber cassumunar* Roxb. is a plant in Zingiberaceae family. It is a widely cultivated medicinal plant and commonly used in traditional medicine as a remedy to alleviate inflammatory disorders in Southeast Asia (Pongprayoon et al. 1997; Bhuiyan et al. 2008; Sukatta et al. 2009). Nonetheless, in order for the rhizomes to be effective, the *Z. cassumunar* plant has to be at least 2 years old. In an effort to circumvent the long waiting time, this study focuses on the leaves of *Z. cassumunar*, which grow much faster than the rhizome and are simpler to collect. This research is the study of the endophytes from the leaves of *Z. cassumunar* and investigate the endophyte extracts on antibacterial and antioxidant activity. Results from this study would provide valuable information for the development of novel pharmaceutical agents.

## Results and discussion

From a well-known medicinal plant *Z. Cassumunar*, the authors have isolated 44 endophytes from several segments of healthy leaves. All of the cultures from the endophytes were deposited at the Center of Excellence in Fungal Research-Mae Fah Luang University to generate MFLUCC codes (MFLUCC16-1045 to 16–1088). The endophytes were identified in the genus level based on the fungal characteristic. Under Leica DMLB and Leitz microscopes, the morphology of the endophytes was characterised based on taxonomically relevant features including a shape of conidia, type of conidiophore, growth rate and colony colour. From the observation, the endophytes were classified in seven genera, which are genus *Arthrinium, Aspergillus, Colletotrichum, Greeneria, Lasiodiplodia* and *Ustilago*. All endophytes contained heavily intertwining hyphae with whitish mycelium. Some cultures from genus *Arthrinium* and *Aspergillus* appeared olive green in colour, but white mycelium appeared after 10 days. Olivaceous brown exudates appeared after 20 days of incubation at room temperature in all isolates. Zingiberaceae is the plant family which is widely distributed throughout in Southeast Asia and it presents significant resistance to both biotic and abiotic stresses (Habsah et al. 2000). *Z. Cassumunar* is the plant in the family Zingiberaceae that is widely used in folklore remedies in many Asian countries. The plant oil from the rhizome extracts has anti-inflammatory and anti-microbial activities (Pongprayoon et al. 1997; Bhuiyan et al. 2008; Sukatta et al. 2009). Recently, several ascomycetous endophytic fungal species were detected in the rhizomes of healthy *Zingiber* plants (Ginting et al. 2013; Jasim et al. 2014; Krishnapura and Belur 2016). These results suggest that the number and diversity of endophytic fungi depend on the geographic location rather the host plant species.

The ethyl acetate extracts of all endophytes were studied on antibacterial activities with gram-negative and gram-positive bacteria (Kamazeri et al. 2012). The most extracts inhibited at least one of the tested bacterial strains (Table 1). The crude extract of *Arthrinium* sp. MFLUCC16-1053 showed antibacterial activity against most cell line tested, for *Escherichia coli* (gram-negative) the MIC was 7.81, same value as the standard. This crude extract had antibacterial activity against most tested human pathogens, but especially against gram-positive bacteria. This may be related to the cell wall structure of gram-negative bacteria, which is comprised of hydrophilic lipo-polysaccharides preventing penetration by hydrophobic oils and diverting accumulation of fungal extracts in the target cell membrane (Bajpai et al. 2011). This result suggested that the ethyl acetate extract of the endophytes from *Z. cassumunar* highly effective to gram-positive bacteria better than gram-negative one. Nonetheless, the extract of *Arthrinium* sp. MFLUCC16-1053 was effective against all gram-negative pathogens used in this study, albeit to a lesser degree. It might be that the extract of *Arthrinium* sp. MFLUCC16-1053 contains the antibacterial compounds. The GC-MS analysis was used to identify volatile compounds of the MFLUCC16-1053 extract. The relative area percentages and their retention indices are summarised in Table 2. β-Cyclocitral which has antibacterial properties is the main component in the extract of *Arthrinium* sp. MFLUCC16-1053 showing peak area at 27.81% (Paun et al. 2013). Other major compounds including 3*E*-cembrene A (8.04%), laurenan-2-one (3.50%) and sclareol (6.26%) were also identified, all of which have been reported to have antibacterial and other pharmacological activities (Chaudhary et al. 2012). Moreover, Nonetheless, minor compounds such as 2*Z*,6*E*-farnesol (1.38%), bicyclogermacrene (1.07%), γ-curcumene (0.77%), cembrene (0.44%) and β-isocomene (0.12%) also display important antibacterial properties (Burt 2004). It is well known that the presence of various bioactive compounds including monoterpenoids, sesquiterpenoids and their derivatives improve the potency and antibacterial properties of fungal extracts (Ramalakshmi and Muthuchelian 2011). However, the extract of *Arthrinium* sp. MFLUCC16-1053 showed low antibacterial activity which might by it contains active components in very low amounts. Antibacterial activity of fungal crude extracts may also be the result of synergistic action of various compounds in the extract rather than major compounds individually.

**Table 1.** Antibacterial activity expressed as inhibition zone diameter (mm) and MIC (μg/mL) of ethyl acetate extracts of the endophytes from *Z. cassumunar* leaves and chloramphenicol.

		MIC, µg/mL (zone diameter, mm±SD)					
		Gram-positive			Gram-negative		
Fungal extract	MFLUCC code 16-	I	II	III	IV	V	VI
*Arthrinium* sp.	1045	–	–	–	–	–	15.62 (8.3 ± 1.3)
*Lasiodiplodia* sp.	1046	31.25 (9.2 ± 1.1)	125 (13.4 ± 1.5)	125 (11.3 ± 2.5)	–	–	31.25 (8.1 ± 2.7)
*Arthrinium* sp.	1047	–	–	–	1000 (12.1 ± 2.7)	–	7.81(10.3 ± 1.8)
*Aspergillus* sp.	1048	–	–	–	1000 (12.1 ± 2.6)	–	7.81 (11.3 ± 3.4)
*Aspergillus* sp.	1049	–	125 (11.1 ± 2.6)	–	1000 (10.3 ± 2.7)	500 (12.1 ± 2.5)	62.50 (11.2 ± 2.2)
*Colletotrichum* sp.	1050	–	–	–	1000 (13.5 ± 2.9)	–	31.25 (8.6 ± 1.8)
*Colletotrichum* sp.	1051	–	125 (8.4 ± 2.5)	125(7.9 ± 2.7)	–	–	–
*Aspergillus* sp.	1052	31.25 (10.2 ± 3.5)	–	125 (7.5 ± 2.4)	–	–	7.81 (11.5 ± 1.9)
***Arthrinium* sp.**	**1053**	**31.25 (9.5 ± 1.9)**	**125 (15.8 ± 2.4)**	**125 (14.4 ± 2.8)**	**1000 (10.5 ± 2.4)**	**125 (15.5 ± 2.9)**	**7.81 (12.8 ± 1.8)**
*Colletotrichum* sp.	1054	–	–	1000 (14.4 ± 3.1)	–	–	62.50 (9.8 ± 2.7)
*Aspergillus* sp.	1055	–	–	–	–	–	–
*Aspergillus* sp.	1056	62.50 (11.4 ± 1.6)	125 (15.5 ± 2.2)	125 (13.4 ± 1.8)	–	–	–
*Colletotrichum* sp.	1057	–	–	125 (9.1 ± 3.2)	–		31.25 (10.4 ± 2.5)
*Greeneria* sp.	1058	–	1000 (8.5 ± 2.4)	–	–	–	–
*Greeneria* sp.	1059	–	–	1000 (9.6 ± 2.1)	–	–	–
*Colletotrichum* sp.	1060	62.50 (8.5 ± 2.4)	–	–	–	–	–
*Colletotrichum* sp.	1061	–	–	–	–	–	–
*Colletotrichum* sp.	1062	–	–	–	–	–	–
*Aspergillus* sp.	1063	–	–	–	–	–	–
*Aspergillus* sp.	1064	–	250 (9.5 ± 1.8)	125 (8.8 ± 2.5)	–	–	–
*Colletotrichum* sp.	1065	–	–	–	–	–	–
*Aspergillus* sp.	1066	–	250 (9.2 ± 2.2)	1000 (9.8 ± 2.5)	–	–	–
*Lasiodiplodia* sp.	1067	–	–	–	–	–	–
*Colletotrichum* sp.	1068	–	–	1000 (7.5 ± 1.2)	–		–
*Aspergillus* sp.	1069	–	–	–	–	–	–
*Fusarium* sp.	1070	–	–	1000 (9.3 ± 1.7)	–	–	31.25 (9.4 ± 2.7)
*Colletotrichum* sp.	1071	31.25 (13.2 ± 3.3)	125 (13.4 ± 2.1)	125 (12.1 ± 1.9)	–	–	–
*Arthrinium* sp.	1072	–	–	1000 (10.4 ± 2.3)	–	500 (12.2 ± 1.4)	31.25 (10.2 ± 2.1)
*Ustilago* sp.	1073	–	1000 (12.1 ± 1.8)	1000 (8.2 ± 1.5)	–	–	–
*Greeneria* sp.	1074	–	–	1000 (14.2 ± 2.1)	–	–	–
*Greeneria* sp.	1075	–	–	–	–	–	–
*Colletotrichum* sp.	1076	–	–	1000 (8.5 ± 1.4)	–	–	–
*Colletotrichum* sp.	1077	–	–	–	–	–	–
*Colletotrichum* sp.	1078	–	500 (8.6 ± 1.8)	500 (9.2 ± 1.7)	–	–	–
*Colletotrichum* sp.	1079	–	–	–	–	–	31.25 (10.1 ± 1.9)
*Fusarium* sp.	1080	62.50 (10.5 ± 2.3)	–	1000 (10.8 ± 2.2)	–	–	31.25 (8.3 ± 1.8)
*Colletotrichum* sp.	1081	–	125 (12.2 ± 2.1)	1000 (10.5 ± 2.3)	–		–
*Colletotrichum* sp.	1082	–	–	–	–	–	–
*Greeneria* sp.	1083	–	1000 (10.6 ± 2.4)	500 (8.4 ± 1.7)	–	–	–
*Greeneria* sp.	1084	–	500 (8.3 ± 2.1)	500 (12.2 ± 2.2)	–	–	–
*Greeneria* sp.	1085	–	–	500 (10.5 ± 2.3)	–	–	–
*Aspergillus* sp.	1086	–	–	–	–	–	–
*Arthrinium* sp.	1087	–	–	–	–	–	–
*Arthrinium* sp.	1088	–	–	1000 (12.5 ± 1.8)	–	1000 (10.2 ± 2.1)	–
chloramphenicol	-	15.62 (12.4 ± 1.4)	15.62 (9.6 ± 2.2)	62.50 (10.6 ± 2.4)	250 (10.4 ± 2.3)	62.50 (12.5 ± 1.5)	7.81 (10.8 ± 1.1)

– is not detected

^I^*S. aureus* ATCC 25923, ^II^*S. epidermidis* ATCC 12228, ^III^*S. agalactiae* DMST 17129, ^IV^*P. mirabilis* DMST 8212, ^V^*S. typhimurium* ATCC 11778 and ^VI^*E. coli* ATCC 25922.

**Table 2.** Volatile constituent and their relative peak area percentage of ethyl acetate extract of *Arthrinium* sp. MFLUCC16-1053.

Peak	RT (min)	Compound	RI*	%Peak area
1	6.67	3-p-menthone	987	0.06
2	8.51	benzene acetaldehyde	1043	0.16
3	9.74	*cis*-vertocitral C	1078	0.37
4	10.68	α-fenchcamphorone	1104	0.35
5	15.45	β-cyclocitral	1220	27.81
6	16.89	linalool acetate	1254	0.09
7	17.57	citronellyl formate	1270	0.44
8	18.32	bornyl acetate	1288	0.08
9	18.92	*E*-cinnamyl alcohol	1302	0.24
10	19.71	dihydro citronellol acetate	1321	0.31
11	21.62	hydrocinnamyl acetate	1366	0.19
12	21.9	linalool isobutanoate	1373	0.08
13	22.53	*Z*-cinnamyl acetate	1388	0.12
14	23.33	β-isocomene	1407	0.12
15	24.47	isoamyl benzoate	1435	0.43
16	25.58	dehydro-aromadendrene	1462	0.21
17	26.37	γ-curcumene	1481	0.77
18	27.15	bicyclogermacrene	1500	1.07
19	27.89	7-epi-α-selinene	1519	0.09
20	28.13	*E*-iso-γ-bisabolene	1525	0.23
21	29.95	caryophyllenyl alcohol	1572	0.22
22	30.21	himachalene epoxide	1578	0.23
23	30.58	allo-cedrol	1588	0.15
24	30.89	longiborneol	1596	0.12
25	31.25	curzerenone	1605	0.21
26	32.36	*cis*-cadin-4-en-7-ol	1635	0.34
27	32.61	α-muurolol	1642	0.18
28	32.82	*Z*-methyl jasmonate	1648	0.30
29	34.09	*trans*-methyl dihydrojasmonate	1682	2.13
30	34.88	amorpha-4,9-dien-2-ol	1703	7.26
31	35.54	2*Z*,6*E*-farnesol	1721	1.38
32	36.13	oplopanone	1737	0.27
33	36.77	α-sinensal	1754	3.14
34	37.91	β-bisabolenol	1785	0.99
35	38.47	epi-α-bisabolol acetate	1800	1.17
36	38.83	β-chenopodiol	1810	0.47
37	39.62	occidol	1831	0.78
38	40.72	*Z,Z*-farnesyl acetone	1861	1.05
39	41.23	*E*-β-santalol acetate	1874	0.70
40	41.87	catalponone	1892	0.16
41	42.51	5*E*,9*E*-farnesyl acetone	1909	0.66
42	43.21	11,12-dihydroxy-valencene	1928	0.41
43	43.52	cembrene	1936	0.44
44	44.01	3*E*-cembrene A	1949	8.04
45	44.86	*Z*-methyl isoprenyl cinnamate	1972	3.86
46	45.68	*Z,E*-geranyl linalool	1994	0.97
47	46.36	13-manool oxide	2013	0.50
48	46.85	*E,E*-geranyl linalool	2026	1.72
49	47.45	4-hydroxy-stilbene	2042	0.33
50	48.31	*E*-methyl isoprenyl cinnamate	2065	0.39
51	49.36	benzyl cinnamate	2094	6.47
52	50.08	laurenan-2-one	2113	3.50
53	51.03	*E*-isoeugenyl benzyl ether	2139	1.19
54	51.35	abienol	2147	0.32
56	52.58	phenethyl cinnamate	2180	0.85
57	53.05	ethyl octadecanoate	2193	2.53
58	53.41	catalpalactone	2201	0.19
59	53.66	phyllocladanol	2205	0.56
60	54.56	*E*-phytol acatate	2217	0.81
61	54.91	sclareol	2221	6.26
62	56.21	7-α-hydroxy manool	2238	0.26
63	56.75	*Z*-isoeugenyl phenyl acetate	2246	0.63

* RI: retention index on HP-5MS column

**Figure 1.**
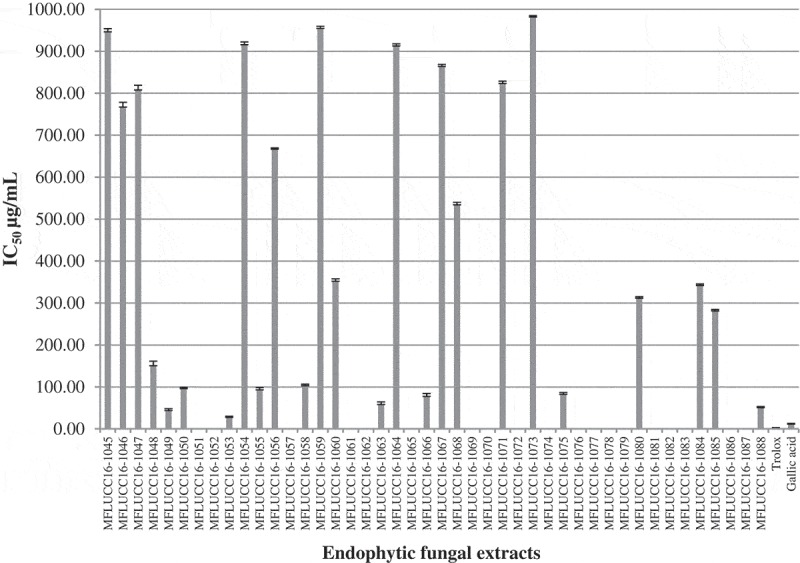
Antioxidant activities of endophytic fungal extracts from *Z. cassumunar* leaves and standard compounds (trolox and gallic acid). Absence of bars denotes no antioxidant activity.

Not only the antibacterial activity, but the ethyl acetate extracts were also studied on the antioxidant activity. The 2,2-diphenyl-1-picrylhydrazyl (DPPH) radical scavenging ability of the extract of *Arthrinium* sp. MFLUCC16-1053 showed higher antioxidant property at IC_50_ of 28.47 µg/mL. Trolox and gallic acid were used as standards (Figure 1). This result suggests that the extract of *Arthrinium* sp. MFLUCC16-1053 is an effective radical scavenger and can be potentially used as an antioxidant supplement. The GC-MS analysis of volatile components in the extract of *Arthrinium* sp. MFLUCC16-1053 contained benzene acetaldehyde, 3-p-menthone, bornyl acetate, γ-curcumene, bicyclogermacrene and β-isocomene, might be responsible to some extent for the notable antioxidant properties of *Arthrinium* sp. (Gonzalez-Burgos and Gomez-Serranillos 2012) due to the presence of strongly activated methylene groups. However, Mata et al. opposed this by reported that extracts containing terpene compounds were considered to have low antioxidant activity in the DPPH reduction due to low solubility in methanol solvent resulting in low capability of donating a hydrogen atom (Mata et al. 2007). Thus, the significant antioxidant activity of the extract of *Arthrinium* sp. MFLUCC16-1053 could be also be the result of other, as yet unidentified phenolic compounds and derivatives or to a synergistic effect of several compounds.

**Figure 2.**
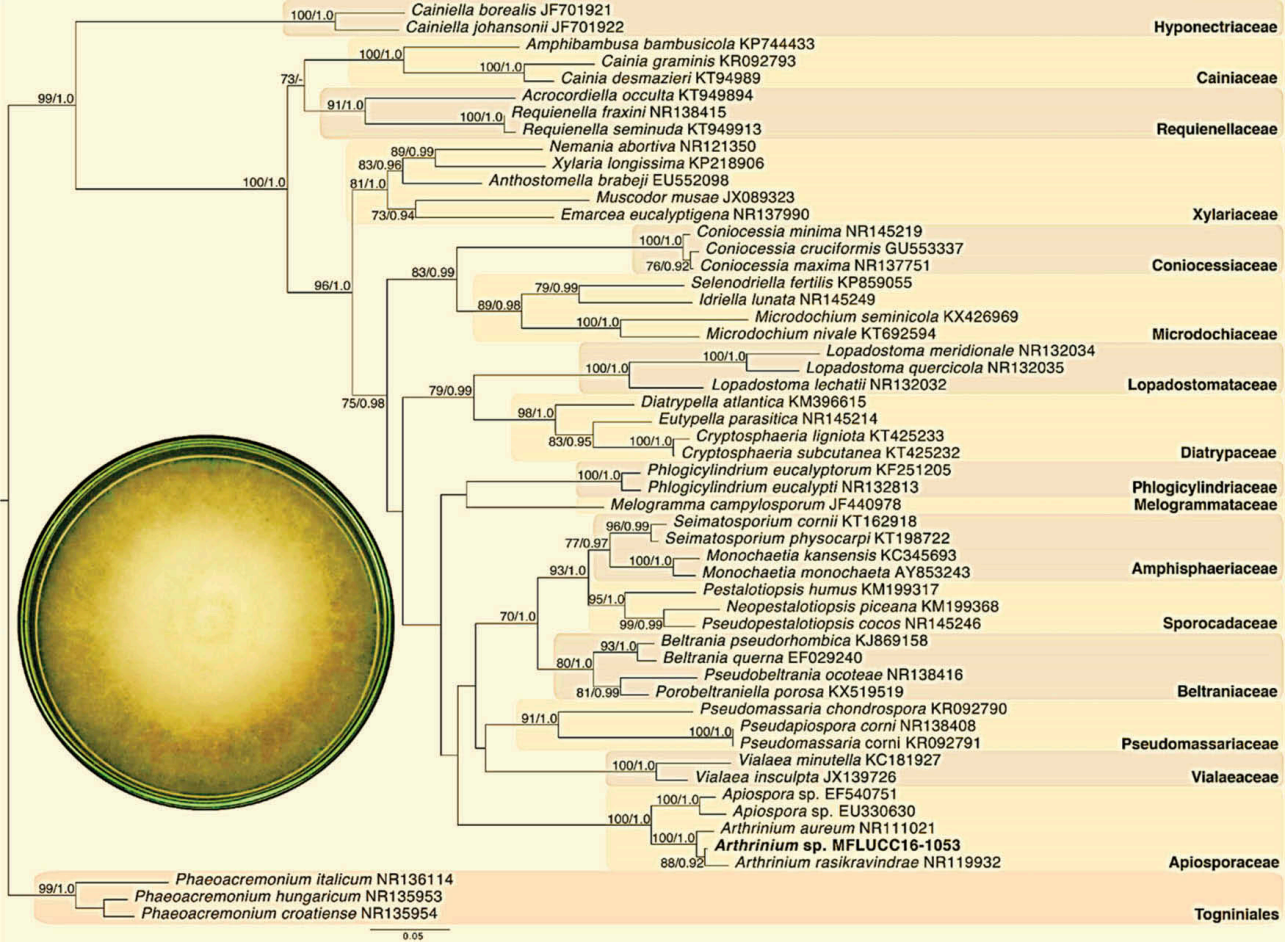
Phylogenetic tree of internal transcribed spacers inferred from 51 taxa and 830 sites under the GTR model of nucleotide substitution + ΓThe newly sequenced endophytic fungus *Arthrinium* sp. MFLUCC16-1053 is in bold. Bootstrap support values for maximum likelihood greater than 70% and Bayesian inference posterior probabilities greater than 0.90 are shown.

The value of combining morphological characters and phylogenetics has been demonstrated repeatedly (Jaklitsch et al. 2016). Morphological features of *Arthrinium* sp. were analysed and compared as previously reported (Adelantado et al. 2005). The colonies of strain MFLUCC16-1053 were black or dark brown with mycelia that were moderately superficial and immersed while fructifications were mostly superficial. The hyphae were narrow and bonded through the host cuticle. Conidiophores were produced from subspheric, ampulliform and barrel-shaped cells. Molecular and phylogenetic methods were carried out to further confirm the identification of the endophytic fungal strain MFLUCC16-1053 isolated from *Z. cassumunar* leaves. The tree topologies derived from the maximum likelihood and Bayesian inference methods were very similar (Figure 2). The newly sequenced endophytic fungus *Arthrinium* sp. MFLUCC16-1053 from *Z. cassumunar* grouped with Apiosporaceae within the genus *Arthrinium* clade with maximum support (100BS/1.0PB) indicating that it does indeed belong to the *Arthrinium* genus.

## Conclusion

The *Arthrinium* sp. MFLUCC16-1053 which is one of the 44 isolated endophytes from *Z. cassumunar* leaves. The ethyl acetate extract of *Arthrinium* sp. MFLUCC16-1053 exhibited various degrees of antibacterial against six human bacterial pathogens. It significantly inhibited on gram-positive bacteria (*S. aureus* ATCC 25923, *S. epidermidis* ATCC 12228 and *S. agalactiae* DMST 17129) better than gram-negative bacteria (*P. mirabilis* DMST 8212, *S. typhimurium* ATCC 11778 and *E. coli* ATCC 25922). Moreover, the extract of *Arthrinium* sp. MFLUCC16-1053 also has antioxidant activity on DPPH radical scavenging assay comparing to the standards (trolox and gallic acid). These experiment suggests that the ethyl acetate extract of *Arthrinium* sp. MFLUCC16-1053 can be exploited as a natural antibiotic against bacterial diseases and as an antioxidant in line with its notable antioxidant and antibacterial properties revealed in this study.

## Materials and methods

### Plant materials

Leaves of 1-year-old *Z. cassumunar* were collected in Thoeng District, Chiang Rai province located in the Northern part of Thailand (100°71ʹ58”E, 19°70ʹ69”N) in May 2016. *Z. cassumunar* was identified morphologically and a voucher herbarium specimen (MFL No. 10003) was deposited at the Mae Fah Luang Botanical Garden, Chiang Rai, Thailand.

### Isolation of endophytic fungi

Healthy leaves of *Z. cassumunar* were washed with distilled water for one min and then air-dried. The leaf surface was sterilised by dipping the leaves in 70% ethanol, followed by dipping in 1% sodium hypochlorite and subsequently rinsing in sterile demineralised distilled water. The cleaned leaves were cut into pieces of approximately 0.5 cm^2^. The leaf layers were plated in Petri dishes containing sterile water agar plates containing chloramphenicol. The plates were incubated at room temperature for three days. The hyphal tip of endophytes growing out from the plant tissues were cut and transferred to new potato dextrose agar (PDA) plates. The plates were stored at room temperature for 7 days. All of the endophyte cultures were deposited at Center of Excellence in Fungal Research Mae Fah Luang University. All isolated endophytic fungi were characterised according to the modified method of Sharma et al. (2016), including microscopic observation of mycelia, asexual/sexual spores and colony morphology.

### Fermentation and extraction

The endophyte cultures on PDA were cut in small pieces (6 mm of diameter) and further transferred into 1000 mL Erlenmeyer flask containing 150 mL of potato dextrose broth (PDB). After incubation at room temperature for 30 days, the fermentation broth of each fungus was vacuum-filtered prior to macerating their mycelia filtrates in 150 mL ethyl acetate at room temperature for 3 days. The mixture was subsequently partitioned in a 500 mL separating funnel and then ethyl acetate solvent (100 mL × 3) was used to extract the remaining PDB culture broth. The ethyl acetate extracts from the mycelia and PDB culture broth were combined and concentrated by using a vacuum rotary evaporator at reduced pressure at 40°C. The concentrated crude extracts were then stored at 4°C to be used for studying the biological activities.

### Antibacterial activity of fungal crude extracts

Six human pathogenic bacteria were used in the screening of the antibacterial activity of the ethyl acetate extracts of all endophytes. They contained both gram-positive (*Staphylococcus aureus* ATCC 25923, *Staphylococcus epidermidis* ATCC 12228, and *Staphylococcus agalactiae* DMST 17129) and gram-negative bacterial strains (*Proteus mirabilis* DMST 8212, *Salmonella typhimurium* ATCC 11778 and *Escherichia coli* ATCC 25922). All of the bacteria in this experiment were obtained from Department of Medical Science, Ministry of Health Bangkok, Thailand while the bacteria with DMST codes were obtained from Culture Collection for Medical Microorganisms, Department of Medical Sciences, Thailand. Before testing, the bacteria strains were sub-cultured in tryptic soy broth medium for 24 h. The agar disc diffusion method was used to investigate the antibacterial activity of all ethyl acetate extracts of endophytes from *Z. cassumunar*. Each crude extract was diluted by using ethyl acetate solvent at 1:1 ratio. The bacterial strains were swabbed on a sterile Petri dish Muller Hinton agar after adjusted to 0.5 McFarland standard. A sterilised 6-mm of a paper disc (Whatman^TM^, USA) containing 30 μL of the ethyl acetate extracts was placed on the infusion agar individually, and then the plates were kept at 37°C for 24 h. Chloramphenicol is used as a positive control in this experiment. All experiments were carried out in triplicate (*n* = 3) and results are reported as mean±SD values.

### Scavenging 2,2-diphenyl-1-picrylhydrazyl (DPPH) free radicals assay

The antioxidant activity of the ethyl acetate extracts was studied on a DPPH radical scavenging activity. The ethyl acetate extracts was dissolved in methanol and mixed with 60 mM methanol solution of DPPH in 1:1 ratio. The mixture solutions were kept in the dark place for 30 min, and then determined by spectrophotometry at 517 nm in a spectrophotometer by using methanol solvent as a blank and the absorbance of the DPPH radical without antioxidant served as the negative control. The amount of sample necessary to decrease the absorbance of DPPH by 50% (IC_50_) was calculated graphically and the percentage inhibition was calculated according to the equation: % inhibition = 100 × [(Absorbance of control–Absorbance of sample)/Absorbance of control]. Both trolox and gallic acid were used as positive controls. All experiments were carried out in triplicate (*n* = 3) and results are reported as mean values.

### GC-MS analysis of volatile components of Arthrinium sp. MFLUCC16-1053

The chemical composition of fungal extract from *Arthrinium* sp. MFLUCC16-1053 was identified using a Hewlett Packard model HP6890 gas chromatograph (Agilent Technologies, Palo Alto, CA, USA) fitted with a HP-5MS (5% phenyl-polymethylsiloxane) capillary column (30 m × 0.25 mm i.d., film thickness 0.25 μm; Agilent Technologies, USA). The instrument was set to an initial temperature of 60°C. At the end of this period the oven temperature rose up to 250°C, with a rate of increase of 3°C/min. The electron multiplier voltage was 1150 V. The ion source and quadrupole temperatures were set at 230 and 250°C, respectively. Helium was used as the carrier gas with a flow rate of 1 mL/min. The ionisation voltage was 70 eV. The samples were injected in split mode as 50:1. Mass spectral scan range was set at 29–300 (m/z). The identification of chemical compounds present in the extract was performed by comparing the mass spectra with data from NIST05 (National Institute of Standards and Technology, US) and WILEY 7N libraries, and comparison of their Kováts retention indices, relative to C_8_–C_23_*n*-alkanes. All identified components were summarised in terms of relative peak area percentage.

### Genomic DNA extraction of Arthrinium sp. MFLUCC16-1053 and PCR

*Arthrinium* sp. MFLUCC16-1053 was selected to complement the morphological identification due to its promising antibacterial and antioxidant activities which were notably higher than the rest endophytic fungal extracts. The aerial mycelium of strain MFLUCC16-1053 was scraped from the PDA surface. This fungal biomass was further pulverised into a fine powder with a pestle and mortar. The genomic DNA of this strain was extracted using cetyltrimethylammonium bromide (CTAB) according to manufacturer’s specifications. CTAB has previously been used to extract nucleic acids from endophytes successfully (Soltani and Moghaddam 2015). To supplement the database with molecular information of the new isolate the barcode fragment ITS1-5.8S-ITS2 sequence was amplified using the universal primers, ITS4 (5ʹ-TCCTCCGCT TATTGATATGC-3ʹ) and ITS5 (5ʹ-GGAAGTAAAAGTCGTAACAAGG-3ʹ). The PCR conditions were: 95°C for 5 min, followed by 40 cycles of 95°C for 50 s, 52°C for 50 s, 72°C for 50 s, and final extension at 72°C for 10 min on a PeqSTAR 2× thermal cycler (Peqlab, Germany). PCR products were checked on 1% agarose gels stained with ethidium bromide under UV light and purified using NucleoSpin® Gel and PCR Clean-up Kit (Macherey-Nagel, Germany). The purified PCR products were directly sequenced in both directions at the 1^ST^ Base Company (Malaysia) using the same PCR primers mentioned above. The acquired gene sequence was submitted to the NCBI Genbank database under an accession number as KY665715.

### Phylogenetic analysis

The newly acquired sequence was subjected to BLAST search against the National Center for Biotechnology Information (NCBI) database to exclude bacterial contamination. Following this, we interrogated the nr database using blastn and assembled our dataset, which contained representative taxa spanning the Xylariomycetidae. Sequences were aligned using MAFFT (default parameters) and trimmed with trimAl, automated option. After trimming, 830 sites remained in the alignment. A maximum likelihood phylogenetic tree was constructed using RAxML-HPC v.8 and the general time reversible model of nucleotide substitution. Bootstrap values were obtained from 1000 replicates. In order to further confirm our phylogenetic hypothesis, a Bayesian inference analysis was also performed, whereby four Monte Carlo Markov chains were run for 1,000,000 generations. Convergence was declared when all RI parameters converged towards zero.
